# Sixteen-Week Vitamin D_3_ Supplementation Increases Peripheral T Cells in Overweight Black Individuals: Post hoc Analysis of a Randomized, Double-Blinded, Placebo-Controlled Trial

**DOI:** 10.3390/nu14193922

**Published:** 2022-09-22

**Authors:** Yutong Dong, Li Chen, Ying Huang, Anas Raed, Robyn Havens, Yanbin Dong, Haidong Zhu

**Affiliations:** 1 Georgia Prevention Institute, Department of Medicine, Medical College of Georgia, Augusta University, Augusta, GA 30912, USA; 2 Internal Medicine Residency Program, Department of Medicine, New York University Langone Health, New York, NY 10016, USA; 3 Department of Medicine, Medical College of Georgia, Augusta University, Augusta, GA 30912, USA; 4 School of Nursing, University of South Carolina, Aiken, SC 29801, USA

**Keywords:** vitamin D, immune system, African American, T cell

## Abstract

Background: Vitamin D is considered to modulate T-cell function, which has been implicated in the treatment of inflammatory conditions. However, there is limited knowledge on the effects of vitamin D and its influences on circulating T-cell profiles in humans, particularly in overweight Black individuals who are more likely to be vitamin D insufficient (serum 25(OH)D concentrations of ≤20 ng/mL). Thus, this study tested the hypothesis that vitamin D supplementation modulates T-cell composition, which is in a dose-dependent manner. Methods: A 16-week randomized, double-blinded, placebo-controlled trial of vitamin D_3_ supplementation was undertaken in 70 overweight/obese Black people (mean age = 26 years, 82% female) with 25 hydroxyvitamin D ≤ 20 ng/mL at baseline. Subjects were randomly assigned a supervised monthly oral vitamin D_3_ equivalent to approximately 600 IU/day (n = 17), 2000 IU/day (n = 18), 4000 IU/day (n = 18), or a placebo (n = 17). Fresh peripheral whole blood was collected and CD3^+^, CD4^+^ and CD8^+^ cell counts and percentages were determined by flow cytometry at baseline and at 16 weeks, among 56 subjects who were included in the analyses. Results: A statistically significant increase in CD3^+^% in the 2000 IU/day vitamin D_3_ supplementation group, and increases in CD4^+^% in the 2000 IU/day and 4000 IU/day vitamin D_3_ supplementation groups were observed (*p*-values < 0.05) from the changes in baseline to 16 weeks. Further adjustments for age, sex and BMI showed that 2000 IU/day vitamin D_3_ supplementation increased in CD3^+^ count, CD3%, CD4 count, and CD4%, as compared to the placebo group (*p*-values < 0.05). Moreover, the highest serum 25(OH)D quantile group had the highest CD3% and CD4%. Conclusions: Sixteen-week vitamin D_3_ supplementation increases peripheral blood T-cell numbers and percentages in overweight/obese Black patients with vitamin D insufficiency. This resulting shift in circulating T-cell composition, particularly the increase in T helper cells (CD4^+^ cells), suggests that vitamin D supplementation may improve immune function in Black individuals.

## 1. Introduction

Low vitamin D status is prevalent worldwide, even in sunny climates such as the US Southeast [1,2,3,4]. Furthermore, the prevalence of vitamin D deficiency in the US is higher among the Black population, particularly those living inside the Stroke Belt, compared to other races [5]. Vitamin D deficiency is a risk factor for weak immunity [6,7,8,9]. Recently, the vitamin D and omega-3 trial (VITAL)—a nationwide, randomized, double-blind, placebo-controlled trial—reported that vitamin D supplementation for five years, with or without omega-3 fatty acids, reduced autoimmune disease by 22% [10]. Mechanistically, vitamin D deficiency can affect our immunity, because vitamin D receptors are expressed on T lymphocytes (T cells) [11]. Thus, vitamin D supplementation has been found to modulate our immune system beneficially, including elevated levels of CD3^+^ and CD4^+^ [12,13,14,15]. As the immune system loses CD4^+^ cells, it becomes weaker. Studies have reported that lower CD4^+^ cell counts contribute to weakened immune systems in HIV patients [16,17]. Another study has suggested that low CD4^+^ together with low CD19^+^ and high CD8^+^ have associations with a higher mortality risk in the non-HIV population [18]. CD3^+^ cells are known to differentiate into CD4^+^ or CD8^+^ cells [19]. However, despite promising results from animal and human studies, prospective studies on the direct effects of vitamin D supplementation on immunity are still very limited [12]. Additionally, given the many benefits of vitamin D, further research has been called to investigate vitamin D in different populations, including high-risk groups [2].

We have previously reported from our randomized, placebo- controlled clinical trial (RCT) that higher doses of vitamin D have improved suboptimal vitamin D statuses in the high-risk group of overweight and obese Black people living inside the Stroke Belt [4]. We investigated whether vitamin D supplementation affects CD3+, CD4+, and CD8+ percentages and cell counts, as surrogate markers of a potentially improved immunity in our high-risk group.

## 2. Materials and Methods

### 2.1. Participants

This randomized, double-blinded, placebo-controlled clinical trial (clinicaltrials.gov registration#: NCT01583621) recruited participants from the community in Augusta, GA, and surrounding areas [4]. All participants had below-normal vitamin D levels (defined as serum 25(OH)D concentrations ≤ 20 ng/mL) at their screening visits [5]. The body mass index (BMI) ≥ 25 kg/m^2^ for adults and ≥85th percentile for adolescent age and sex was used as the criterion to define overweight/obese according to the Centers for Disease Control and Prevention (CDC) criteria. One hundred and twenty-nine overweight/obese Black individuals were telephone-screened for eligibility, with 70 eligible subjects enrolled and followed-up between December 2011 and November 2012. A total of 56 subjects with a T-cell profile were included in this study. The institutional review board (IRB) at Augusta University approved this study (IRBNet ID #611339). Informed written assent and consent were obtained from adolescents and their guardians, respectively, with adults providing informed consent.

### 2.2. Study Design

The participants were randomly assigned to any one of the four groups of 18,000 IU/month (~600 IU/day), 60,000 IU/month (~2000 IU/day), 120,000 IU/month (~4000 IU/day) of vitamin D_3_, or a placebo. The interventional capsules were provided to the participants by supervised dosing every 4 weeks at their study visit for 16 weeks to maximize compliance. Bio-Tech Pharmacal, Fayetteville, AR, provided the vitamin D_3_ and placebo capsules, while the Augusta University (AU) clinical research pharmacy generated the randomization codes and dispensed the study capsules. The AU clinical pharmacy marinated the randomized codes until the end of the study and did not have any direct role in the data collection. The sample size determination has been described in a previously published study [4].

### 2.3. Anthropometry Measurements

Height and weight were obtained according to standard procedures, using a wall-mounted stadiometer (Tanita Corporation of American, Arlington Heights, IL, USA) and a calibrated electronic scale (model CN2OL; Cardinal Detecto, Webb City, MO, USA). Prior to each weekly testing, the accuracy of the electronic scale was checked using known weights. BMI was calculated as weight (kg) divided by height (m^2^).

### 2.4. Biochemical Measurements

Fasting blood samples and spot urine samples were obtained at the baseline, 8 and 16 weeks. They were then frozen and stored at −80 °C until assayed. Serum 25(OH)D concentrations were measured using enzyme immunoassay (Immunodiagnostic Systems, Fountain Hills, AZ, USA). The intra- and inter-assay coefficients of variation (CV) were 5.6 and 6.6%, respectively. Our laboratory is certified by the vitamin D external quality assessment scheme (DEQAS), an international program monitoring accuracy of 25(OH)D measurements. Peripheral blood was collected and sent to the clinical pathology core lab at Augusta University Medical Center within 2 h for the complete blood counts with differentials, which included the total leukocyte counts and percentages of peripheral blood cell types including neutrophils, lymphocytes, monocytes, eosinophils, and basophils. Immune T cell profile (CD3^+^, CD4^+^, and CD8^+^) was performed using flow cytometry.

### 2.5. Statistical Analysis

Descriptive statistics for variables are presented as means with standard deviation. Prior to analysis, Levene’s test was used to check for homogeneity of variances for all variables. One-way analysis of covariance (ANOVA) was used to compare baseline characteristics across the groups. A Pearson chi-squared test was used to test the difference in sex distribution across the intervention groups. Significance was determined with *p*-values less than 0.05.

ANOVA was used to compare the outcomes (CD3^+^%, CD3^+^ count, CD4^+^%, CD4^+^ count, CD8^+^%, and CD8^+^ count) after 16 weeks of intervention among the groups. Two models were used, where the base model was only adjusted for baseline values, and the second model was adjusted for baseline values, age, sex, BMI, and seasons of the year. Results are presented as adjusted outcome values. Post hoc pairwise analyses, after adjustments for baseline values, age, sex, BMI, and seasons of the year, tested differences in the changes from baselines in CD3^+^%, CD3^+^ count, CD4^+^%, and CD4^+^ count among all four groups. The results are graphed in a boxplot with the medians shown in the graphs and the means described below each group. SPSS–IBM Software was used for all statistical analyses (version 24.0 SPSS Inc., Chicago, IL, USA) with the significance level set at α = 0.05.

## 3. Results

### 3.1. General Demographics

The baseline characteristics of our participants (n = 56) are presented in Table 1. There were no significant differences among the four groups regarding age, sex, BMI, and baseline serum 25(OH)D and T cells (*p*-values > 0.05). As expected, higher doses of vitamin D_3_ supplements resulted in greater increases in serum 25(OH)D concentrations, as we have previously reported [4]. No changes in BMI were identified.

### 3.2. Effects of Vitamin D_3_ Supplementation on Serum 25(OH)D

As previously reported, an overall group by time interaction was identified, which suggested dose- and time-dependent increases in serum 25(OH)D concentrations compared to monthly vitamin D supplements (*p* < 0.01) [4,20]. Both 2000 IU and 4000 IU vitamin D groups increased mean 25(OH)D concentrations to 30.50 ± 2.1 and 35.66 ± 3.4 ng/mL, respectively, at 8 weeks, while maintaining similar levels (36.01 ± 3.1 and 34.80 ± 2.4 ng/mL, respectively) at 16 weeks. Post hoc comparisons showed that 25(OH)D concentration changes were significantly higher in the 4000 IU group vs. the 2000 IU group after 8 weeks, but not after 16 weeks (*p* = 0.061).

### 3.3. Changes in T Cells after Vitamin D_3_ Supplementation

**Figure 1:**
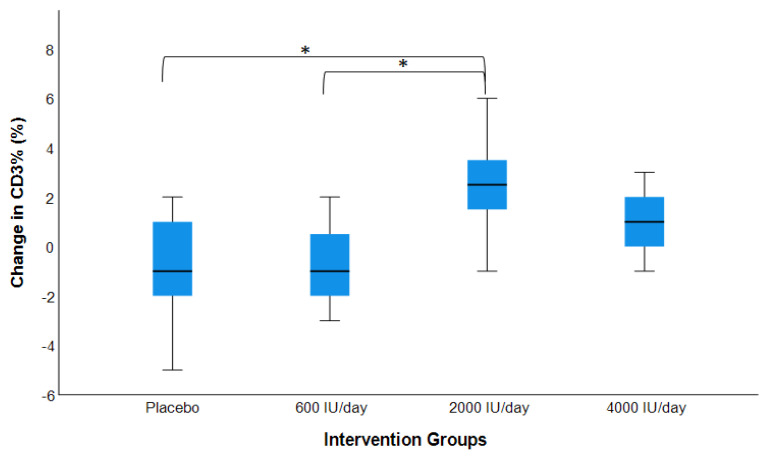
Changes in CD^+^3% (post- vs. pre-intervention values) by dose–response. Model adjusted for baseline value, age, sex, BMI, and seasons of the year (n = 50). * The mean change for the 2000 IU/day group was significantly higher than those for the placebo and 600 IU/day group (*p* < 0.05). No significant differences were found between the 2000 and 4000 IU/day groups.

**Figure 2:**
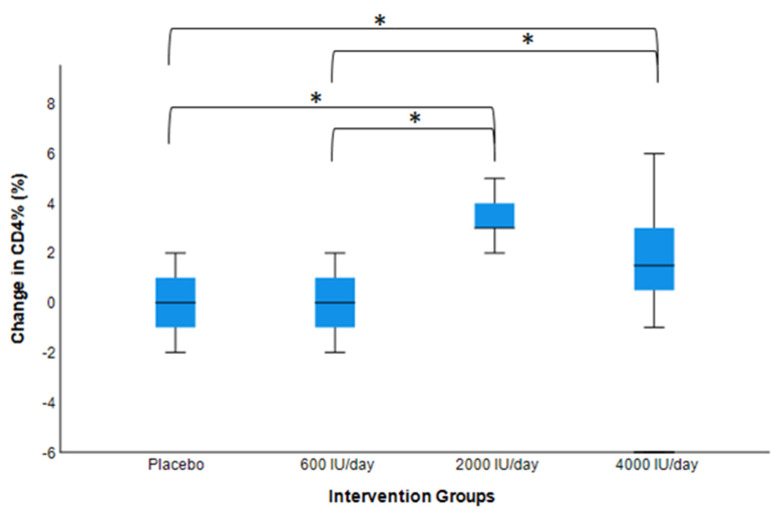
Changes in CD4^+^% (post- vs. pre-intervention values) by dose–response. Model adjusted for baseline value, age, sex, BMI, and seasons of the year (n = 50). * The mean change for the 2000 IU/day group was significantly higher than those for the placebo and 600 IU/day group (*p* < 0.05). No significant differences were found between the 2000 and 4000 IU/day groups.

As shown in Figure 1 and Figure 2, over 16 weeks of vitamin D_3_ supplementation, a statistically significant increase in CD3^+^% in the 2000 IU/day group and increases in CD4^+^% in the 2000 IU/day and 4000 IU/day groups were observed (*p*s < 0.05). Changes in the CD4^+^/CD8^+^ ratio approached significance in the 2000 IU/day group (*p* = 0.05).

**Figure 3:**
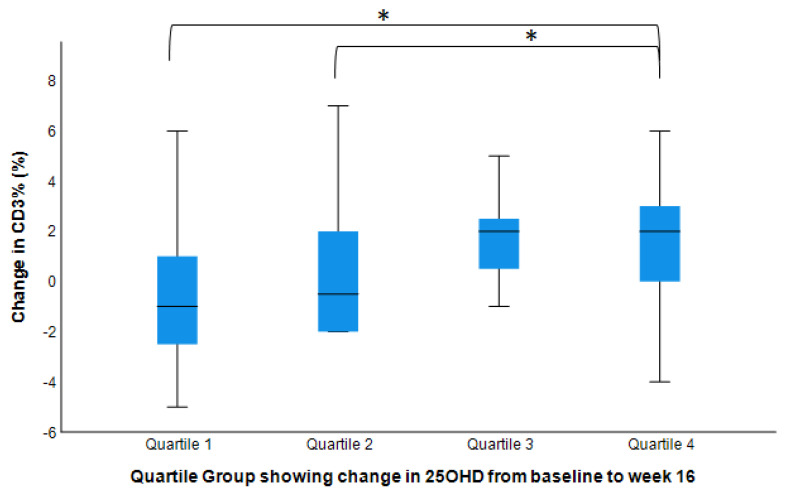
Changes in CD3^+^% by serum 25(OH)D changes (post- vs. pre-intervention values). Model adjusted for baseline value, age, sex, BMI, and seasons of the year (n = 50). Bar plot shows median with IQR and standard deviation from mean. Group 1 had the lowest quartile delta change in 25OHD and Group 4 had the highest. * Mean change for Group 4 was significantly different from Groups 1 and 2, but not with Group 3 (*p*-values < 0.05).

**Figure 4:**
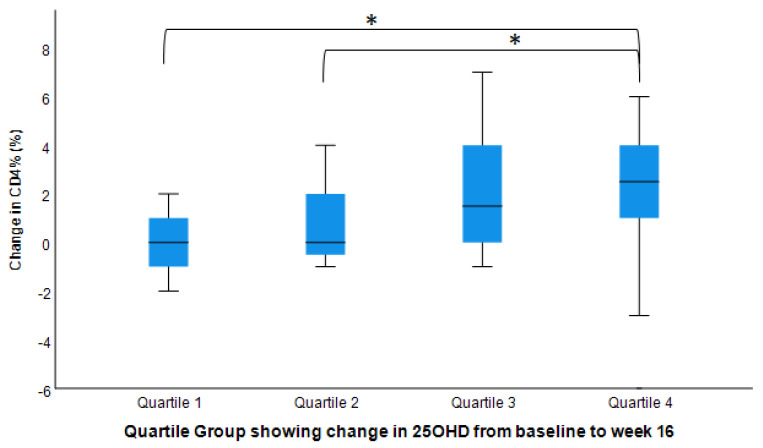
Changes in CD4^+^% by serum 25(OH)D changes (post- vs. pre-intervention values). Model adjusted for baseline value, age, sex, BMI, and seasons of the year (n = 50). Bar plot shows medians with IQR and standard deviations from mean. Group 1 had the lowest quartile delta change in 25OHD and Group 4 had the highest. * Mean change for Group 4 was significantly different from Groups 1 and 2 but not Group 3 (*p* < 0.05).

We also examined the relationship between changes in CD% and changes in serum 25(OH)D, and showed that the highest Delta 25(OH)D quartile group had the highest change in CD3% and CD4% (Figure 3 and Figure 4).

### 3.4. Adjusted Effects of Vitamin D_3_ Supplementation on T Cells

After controlling for sex, age, BMI, and seasons, the clinical intent-to-treat model identified that the different dosage groups of vitamin D_3_ supplements were independent contributing factors to the changes in CD3^+^%, CD4^+^%, and CD4^+^ count (*p*-values < 0.05). Vitamin D_3_ doses were not a significant contributing factor to CD3^+^ count, CD8^+^%, and CD8^+^ count. For the placebo group and 600 IU/day vitamin D_3_ supplementation group, there was a decrease in CD3^+^%, CD3^+^ count, and CD4^+^ count, while there was an increase for the 2000 IU/day and 4000 IU/day supplementation groups. All groups demonstrated an increase in CD4^+^% (*p*-value < 0.05) (Table 2).

### 3.5. Post hoc Pairwise Analysis: Post-Interventional Changes in CD3^+^, CD4^+^, and CD8^+^ by Dosage Groups

In the post hoc group-wise comparison at the end of the 16-week intervention, the changes for the 2000 IU/day vitamin D_3_ supplementation group were only significant when compared to the changes in all other groups. The 2000 IU/day supplementation group resulted in greater increases in CD3^+^% and CD4^+^ count compared to the placebo and 600 IU/day group (*p* < 0.05). The increases in CD3^+^% and CD4^+^ count for the 2000 IU/day supplementation group were greater than the increases for the 4000 IU/day supplementation group, but were not statistically significant. The 2000 IU/day supplementation group also resulted in greater increases in CD3^+^ count compared to the placebo group (*p* < 0.05). Finally, the 2000 IU/day supplementation group had significantly greater increases in CD4^+^% compared to all other groups (*p* < 0.05). No significant changes were found in CD8^+^% nor count in a group-wise comparison.

## 4. Discussion

Vitamin D modulates the human immune system, which may affect our susceptibility to chronic diseases. Although observational studies have identified associations between vitamin D deficiency and chronic immune-associated disorders, prospective studies on the direct effects of vitamin D supplementation on immune cells are extremely limited. To the best of our knowledge, this is the first prospective study on overweight and obese individuals as well as on Black individuals, investigating different dosages of vitamin D supplements and their associated changes in immunity. Our study observed that higher doses (2000 IU and 4000 IU) of vitamin D might improve CD3%, CD4%, and CD4^+^ count with no associated changes in CD8^+^% nor count. Moreover, the highest 25(OH)D quantile group had the highest CD3^+^% and CD4^+^%. Our results provide direct evidence for the possible benefits on immune cells of vitamin D supplementation at high doses, which may result in increased CD3^+^ and CD4^+^ T cells.

The average CD3^+^, CD4^+^, and CD8^+^ percentages and cell counts among our subjects fell within the normal ranges of US adults [21,22]. Overall, the baseline (pre-interventional) average percentages of CD3^+^, CD4^+^, and CD8^+^ cell counts were similar across the different dosage groups. Organizations, including the CDC and the Department of Health and Human Services, have recommended using percentages as a more stable assessment of immune strength compared to cell counts [21,23].

Many studies have demonstrated that vitamin D deficiency is a worldwide epidemic, perhaps even across all ages [1,2,3,5]. Since vitamin D may be crucial to activating our immune defenses, the large prevalence of its deficiency highlights the urgency to investigate the effectiveness and dosage of its supplementation [24].

We found that vitamin D supplements increased CD3+%, CD4^+^%, and CD4^+^ count. Since CD3^+^ helps activate both CD4^+^ and CD8+ T cells, and we observed increase in both CD3^+^ and CD4^+^ percentages, but not in CD8^+^ T cells. Our results suggest that vitamin D supplements seemingly up-regulated CD4^+^, but not CD8^+^ cells [25]. Higher CD4^+^ levels, in general, are associated with reduced risks for immune-mediated inflammatory diseases [26,27,28]. Observational studies have identified that vitamin D deficiency is associated with increased risks for infections, and signs of weakened immunity [29]. Vitamin D insufficiency may also impair our immunity [24]. An in vitro study suggests that the expression of vitamin D receptors plays a crucial role in activating T cells [30]. Mechanistically, because vitamin D receptors are expressed on T cells, vitamin D may have important roles in activating and regulating both memory and naïve T cells, affecting our innate and adaptive immune systems [29,30,31]. In fact, a review paper has concluded that vitamin D can downregulate pathogenic T cells and cytokines and upregulate T_reg_, improving the overall health of our immune system [32]. Our results add support to the growing evidence for the possible therapeutic effects of vitamin D supplementation on immunity and immune-mediated disorders, specifically in overweight and obese Black patients.

The strengths of our study include exclusively recruiting overweight and obese—but otherwise healthy—Black individuals with suboptimal vitamin D status to minimize the confounding effects of weight, race, and disease. The doses of vitamin D selected were based on the current recommended daily allowance (RDA) and tolerable upper intake level (UL) by the Institute of Medicine (IOM). To ensure 100% compliance, a monthly supervised dosing scheme was undertaken [33]. The limitations in our study should also be recognized. Firstly, the percentage of female participation was higher in our sample compared to that of males. However, the sex ratio was similar among the groups. The results also did not differ after adjusting for sex as a potential confounder. Secondly, to ensure the capturing of the variability of vitamin D levels and sunlight, the participants were recruited in different seasons. Only individuals with suboptimal vitamin D levels were recruited, irrespective of the season of enrollment. The results were not changed after adjusting for the seasons. Third, the sample size in each group was relatively small, emphasizing the need for larger studies.

## 5. Conclusions

We found that high-dose vitamin D supplements increased overall CD3^+^ percentage and CD4^+^ percentage and cell counts, but did not affect CD8^+^ percentage and count in overweight and obese Black individuals living inside the Stroke Belt. These findings suggest the potential therapeutic benefits of vitamin D supplements on our immune health. Larger vitamin D dose–response trials on T-cell immunity are warranted.

## Figures and Tables

**Table 1:** Baseline characteristics by intervention groups.

Characteristics	Intervention Groups				*p*-Value
	Placebo	600 IU/Day	2000 IU/Day	4000 IU/Day	
Number of subjects (n)	13	14	15	14	
Age (year)	30 ± 11	27 ± 10	25 ± 8.3	23 ± 6.9	0.23
Male/Female Ratio	4/9	2/12	2/13	1/13	0.39
Height (m)	1.64 ± 0.08	1.65 ± 0.08	1.63 ± 0.09	1.63 ± 0.08	0.83
Weight (kg)	99 ± 26	95 ± 15	92 ± 18	91 ± 21	0.73
BMI (kg/m^2^)	37 ± 7.6	35 ± 5.8	35 ± 7	34 ± 7.7	0.81
Serum 25(OH)D (ng/mL)	35 ± 10	33 ± 9.1	35 ± 9.9	32 ± 10	0.89
T Cells					
CD3^+^%	75 ± 6.7	76 ± 4.2	75 ± 6.8	76 ± 5.6	0.97
CD3^+^ Count (cells/uL)	1533 ± 515	1214 ± 271	1344 ± 454	1452 ± 536	0.30
CD4^+^%	44 ± 5.9	48 ± 7.2	45 ± 6.2	47 ± 7.0	0.49
CD4^+^ Count (cells/uL)	911 ± 321	767 ± 227	812 ± 290	885 ± 298	0.54
CD8^+^%	27 ± 8.5	25 ± 6.4	26 ± 3.7	25 ± 7.4	0.87
CD8^+^ Count (cells/uL)	561 ± 252	390 ± 126	467 ± 171	509 ± 311	0.25

Values for categorical variables are given as numbers (percentages); values for continuous variables as means ± SD.

**Table 2:** Associations between vitamin D supplementation and T cells.

T Cell	Intervention Groups				
	Placebo	600 IU/Day	2000 IU/Day	4000 IU/Day	*p*
CD3^+^% (%)					
Model 1	76 (74, 77)	76 (74, 78)	78 (76, 80)	77 (75, 79)	0.01
Model 2	76 (74, 77)	76 (74, 78)	78 (76, 80)	77 (75, 79)	0.02
CD3^+^ Count (cells/uL)					
Model 1	1375 (1258, 1492)	1382 (1185, 1579)	1555 (1350, 1761)	1469 (1280, 1658)	0.18
Model 2	1384 (1272, 1496)	1388 (1194, 1582)	1552 (1349, 1755)	1468 (1282, 1654)	0.25
CD4^+^% (%)					
Model 1	46 (45, 48)	47 (45, 49)	49 (47, 52)	47 (45, 50)	<0.001
Model 2	47 (45, 48)	47 (45, 49)	50 (47, 52)	47 (46, 50)	<0.001
CD4^+^ Count (cells/uL)					
Model 1	841 (769, 914)	798 (661, 936)	994 (851, 1138)	917 (784, 1049)	0.05
Model 2	847 (781, 914)	804 (669, 939)	995 (854, 1136)	913 (784, 1043)	0.08
CD8^+^% (%)					
Model 1	26 (25, 28)	26 (24, 28)	26 (24, 28)	26 (24, 28)	0.57
Model 2	26 (25, 28)	26 (24, 28)	26 (24, 28)	26 (24, 28)	0.42
CD8^+^ Count (cells/uL)					
Model 1	474 (417, 531)	482 (398, 565)	510 (422, 597)	490 (409, 571)	0.80
Model 2	476 (421, 532)	481 (401, 561)	506 (422, 590)	493 (416, 570)	0.85

Note: Mean estimates represented by mean (95% confidence interval). Model 1 adjusted for baseline value. Model 2 adjusted for baseline value, age, sex, and BMI.

## Data Availability

Data is available upon request.

## Abbreviations

RCT: randomized controlled trial; BMI: body mass index; CDC: Centers for Disease Control and Prevention; IRB: institutional review board; CV: coefficient of variation; DEQAS: vitamin D external quality assessment scheme; ANOVA: analysis of covariance.
